# Two sides of the same coin: the dual effect of star inventors on team innovation

**DOI:** 10.3389/fpsyg.2025.1669363

**Published:** 2026-01-06

**Authors:** Dehong Li, Fang Cui, Yanan Li

**Affiliations:** 1 School of Economics and Management, Chang’an University, Xi’an, China; 2 International Business School, Shaanxi Normal University, Xi’an, China; 3 School of Accounting, Xijing University, Xi’an, China

**Keywords:** innovation, star inventors, team collaboration, technological impact, technological novelty

## Abstract

The scarce yet exceptionally productive star inventors are the pivotal human capital of enterprises. Managers emphasize team-level contributions of star inventors over their individual excellence. However, the impact of star inventors on team innovation remains contested with empirical evidence reporting both positive and negative effects. By theoretically analyzing the advantages and limitations of star inventors at different innovation phases, this study endeavors to reconcile the ongoing debate by investigating the dual effects of star inventors on the novelty and impact of team innovation output. Furthermore, by incorporating technological turbulence and internal network cohesion as moderators, this research demonstrates how external and internal contextual factors shape star inventors’ influence. The findings reveal that star inventors enhance the technological impact of team innovation but hinder its novelty. Technological turbulence weakens their positive effect on impact while exacerbating their negative effect on novelty. Conversely, internal network cohesion amplifies their beneficial influence on impact and mitigates their adverse effect on novelty. By integrating psychological, knowledge management, and social network theories, this study advances the understanding of star inventors’ dual effects and their boundary conditions.

## 1 Introduction

Star inventors, the minority who make disproportionate contributions, are widely recognized as crucial human capital for maintaining the competitiveness of enterprises (Goetze, 2010; Tzabbar and Kehoe, 2014; Liu et al., 2018; Kehoe et al., 2018). As knowledge complexity increases, innovation tends to become collaborative (Wuchty et al., 2007; He et al., 2009; Bernerth et al., 2023; Byron et al., 2023). While these inventors are undeniably stars based on their individual achievements, their roles in team-based innovation remain theoretically contested. Some studies highlight star inventors as valuable repositories of experience and resources that enhance team performance (Ernst and Vitt, 2000; Kehoe and Tzabbar, 2015; Arroyabe et al., 2020), while others demonstrate how their presence can inhibit teammate contributions and lead to adverse collective outcomes (Call et al., 2015; Li et al., 2020). Understanding how teams with star inventors perform systematically is essential for managers to maximize the value of these inventors and allocate R&D resources efficiently (Call et al., 2024). The above-mentioned theoretical tension creates significant challenges for effectively managing innovation teams in practice.

To resolve this contradiction, we argue that innovation must be understood as a multi-stage process where stars’ advantages and limitations manifest differently across phases (Singh and Fleming, 2010; Perry-Smith and Mannucci, 2017). These differential effects are further reflected in distinct dimensions of innovation outcomes. While most existing research predominantly relies on patent citations to evaluate innovation performance, such a unilateral focus likely contributes to the mixed findings in the literature. We contend that a comprehensive assessment should encompass multiple dimensions, as excellence in one dimension may come at the expense of another (Lee et al., 2015; Wagner et al., 2019). Specifically, by examining both technological impact (an invention’s influence on subsequent technological developments) and technological novelty (its degree of novelty relative to existing knowledge), we can more systematically reveal the dual nature of star inventors’ effects on team innovation.

**Figure 1:**
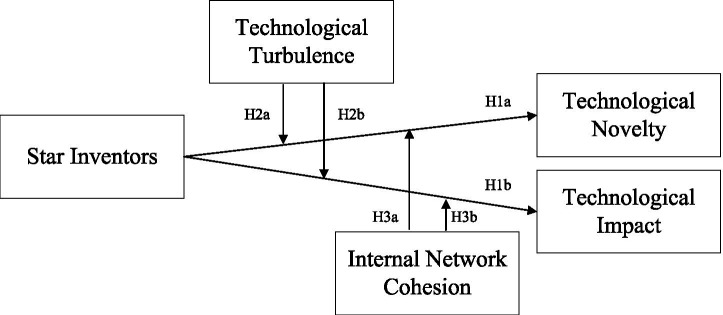
Research framework.

Moreover, team innovation is fundamentally a social process that requires interaction among team members and with the external environment (Li et al., 2020; Khanna and Guler, 2022; Hendricks et al., 2023; Vural and Schillebeeckx, 2025). External and internal contextual conditions can affect the behavior of star inventors and the corresponding consequences. Building on social network and innovation literature (Paruchuri, 2010; Candi et al., 2013; Paruchuri and Awate, 2017; Chen et al., 2018; Dean et al., 2022), we introduce two boundary conditions that shape stars’ effect on team innovation: technological turbulence and internal network cohesion. Technological turbulence refers to the rate of technological change in a specific industry (Dai et al., 2018; Yun et al., 2019). Internal network cohesion refers to the degree of connectivity and inclusiveness among team members (Coleman, 1988; Li et al., 2018). Through this theoretical framework, we systematically examine stars’ dual effects on team innovation, offering managers suggestions for leveraging star talent while inspiring team-level innovative capacity. The framework of this research is shown in Figure 1.

Specifically, based on the common approach in patent-based research where all inventors listed on a patent are treated as a collaborative team (Wuchty et al., 2007; Singh and Fleming, 2010), we empirically examine how the presence of star inventors affects both the impact and novelty of innovation output, while also investigating the moderating roles of technological turbulence and internal network cohesion. The results show that star inventors are indeed beneficial for strengthening the technological impact of innovation output. However, star inventors may potentially compromise the technological novelty of innovation output due to the trap of empiricism and their tendency to maintain their own status. What’s more, a turbulent technological environment can worsen stars’ influence on technological impact, whether positive or negative. In contrast, a cohesive internal network structure leads to their impact evolving in a positive direction.

Our research makes three potential contributions. First, this study extends the understanding of star inventors by revealing their dual roles played in promoting technological novelty and technological impact of team innovation. This provides a possible perspective for addressing the ongoing debate on whether star inventors have a positive or negative impact on team innovation. Second, this research contributes to enriching the contextual factors about the cross-level effect of star inventors on team innovation. Third, by employing the social capital theory and cognitive entrenchment perspective to study the behavior of star inventors at different phases of innovation, this research takes one step forward in bridging psychology and innovation management.

The rest of this research is organized as follows. Section 2 presents the theoretical logic and hypotheses. Section 3 presents a brief introduction to patent data. Section 4 presents methodology, including variable measurement and model specification. Section 5 shows empirical results. Discussion and conclusions are finally provided in Section 6.

## 2 Theory and hypotheses

### 2.1 Theoretical background

#### 2.1.1 Star inventors

Star inventors are recognized as a small elite who produce a disproportionately large share of innovative outcomes, a pattern consistently observed in the production of scientific and technological knowledge (Wuchty et al., 2007; Singh and Fleming, 2010). They are specifically characterized within the innovation literature by “a large number of patent applications of high quality” (Ernst and Vitt, 2000; Ernst, 2001). This exceptional output establishes them as critical human capital, attracting sustained scholarly attention (Kehoe and Tzabbar, 2015; Hohberger, 2016; Lahiri et al., 2019; Call et al., 2024). Subsequent research has refined the understanding of stars by identifying different types, such as those distinguished by performance, social relations, or status (Kehoe et al., 2018). Despite this typological diversity, the fundamental and common criterion for identifying star inventors remains their disproportionate innovative contribution (Arroyabe et al., 2020). Therefore, aligning with the dominant convention in innovation studies, this study adopts the performance-centric definition rooted in the work of Ernst (2001). We operationalize this definition using the dual metrics of patent output quantity and quality, a widely employed method for identifying star inventors (Goetze, 2010; Liu et al., 2018). It should be noted that an early-career inventor who has not yet achieved a sufficient level of output quantity and quality would not be classified as a star inventor in our study. In other words, while an individual may be considered a “rising star” with high potential, they do not meet the specific performance-based threshold required to be defined as a star inventor in this research context.

As summarized in the conceptual review by Call et al. (2015), star inventors are typically characterized by three key attributes: high (1) performance, (2) social capital, and (3) visibility. High performance not only reflects their exceptional output but also implies the accumulation of substantial invention experience. Extensive social capital provides them with access to diverse resources and enhances their social reputation. High visibility ensures that their opinions and outputs are more widely recognized and disseminated (Kehoe and Tzabbar, 2015; Prato and Ferraro, 2018; Li et al., 2020). It should be noted that these attributes of star inventors present both an advantage and a constraint. For example, while accumulated social capital and reputation secure resource support for innovation, they may also induce conservatism, thereby hindering creativity (Bunderson and Reagans, 2011). Focusing solely on one dimension of their influence leads to biased and inconsistent findings.

#### 2.1.2 Phases of innovation

Perry-Smith and Mannucci (2017) have emphasized the importance of clarifying the distinctions among the phases of innovation and understanding the primary needs of each phase to address similar inconsistencies and reconcile previous research. By integrating their approach to conceptualize the phases of innovation with a similar framework proposed by Singh and Fleming (2010), we utilize three phases – idea generation, idea development, and idea implementation – to model the process of innovation. Meanwhile, cognitive flexibility (Amabile, 1983; Dane, 2010), support and influence (Harrison and Rouse, 2015), and shared vision (Obstfeld, 2005) are identified as the primary qualities required from inventors at each stage. Indeed, the characteristics of stars’ behavior that are beneficial for one phase are likely detrimental for another.

### 2.2 Hypotheses

#### 2.2.1 The direct effect of star inventors on technological novelty and impact

The generation of creative ideas is regarded as the starting point of innovation (Koc and Ceylan, 2007; Singh and Fleming, 2010; Perry-Smith and Mannucci, 2017). The novel idea generated at this phase is merely a vague concept, which will be further developed and implemented at the later phases, rather than a comprehensive solution (Perry-Smith and Mannucci, 2017). Although the iterative refinement of creative ideas is a social-psychological process under the context of team collaboration (Vural and Schillebeeckx, 2025), the embryonic form of creative ideas is embedded within a single mind (Singh and Fleming, 2010). It is for this reason that creativity scholars suggest that the flexibility in cognitive structures, rather than the accumulation of mere knowledge and resources, is the essential requirement for generating creative ideas (Amabile, 1983; Dane, 2010; Perry-Smith and Mannucci, 2017). This phase critically shapes the technological novelty of innovation output. However, we argue that involvement of stars is not particularly beneficial for fostering cognitive flexibility at the team level.

Compared to ordinary inventors, star inventors face greater cognitive and social constraints that limit their cognitive flexibility during idea generation.

The cognitive entrenchment perspective suggests that “as one acquires domain expertise, one loses flexibility with regard to problem-solving, adaptation, and creative idea generation” (Dane, 2010). The constraining role of prior experience is powerfully illustrated in Ward’s (1994) cognitive experiment. Faced with the task of imagining and drawing an alien from another planet, participants defaulted to features of known Earth animals, failing to break free from existing cognitive templates. Aligning with this experiment, Levinthal and March (1993) posited that individuals, particularly those with extensive experience, display a strong inclination to apply familiar solutions instead of exploring novel alternatives. Audia and Goncalo (2007) provided direct evidence that an individual’s past success in creative endeavors is negatively related to the future generation of divergent ideas. Star inventors have established strong routines through extensive innovation experience, such that their deep expertise makes it more difficult for them to think beyond established frameworks and adapt to new rules or changes within their domain (Phelps et al., 2012). In contrast, non-star inventors, whose expertise may be less consolidated, may consequently possess an advantage in thinking beyond established frameworks (Phene et al., 2006). As a result, star inventors are more likely to fall into cognitive traps and face the dilemma of cognitive entrenchment.

Beyond cognitive factors, star inventors face more pronounced constraints from their social capital than non-star inventors. Social capital theory suggests that networks, reputation, and status are double-edged: they provide resources but also impose social constraints and encourage risk aversion (Coleman, 1988). For star inventors, their elevated reputation represents a massive sunk cost. Any failure, particularly from exploratory and uncertain ventures, threatens not only the immediate task but also the value of this accumulated reputational capital, likely leading to disproportionate losses compared to their non-star peers (Rhee and Haunschild, 2006). Consequently, stars are rationally motivated to protect this asset by preferring familiar, low-risk solutions over radical explorations (Conti et al., 2014). This strategic conservatism, rooted in protecting social capital, directly dampens their willingness to make the risky cognitive leaps necessary for high novelty, creating a cycle where social constraints reinforce cognitive inflexibility (Perry-Smith and Mannucci, 2017; Li et al., 2018). In contrast, non-star inventors carry less reputational baggage and thus have fewer such constraints. They often possess a stronger motivation to enhance their social standing by challenging and altering existing technological trajectories.

Moreover, the disproportionate influence of star inventors within teams critically amplifies their personal limitations. Due to their outstanding previous performance, star inventors typically occupy central positions in team networks, granting them greater influence over team discourse and decision-making (Nerkar and Paruchuri, 2005; Li et al., 2020). Their suggestions are more likely to be disseminated and accepted. It’s as if an amplifier is installed for star inventors. This implies that whether positive or negative, the influence exerted by star inventors on the team is greater than that of non-stars. The combination of stronger personal constraints (cognitive and social) and greater structural influence means that the presence of a star inventor is more likely to restrict the team’s collective opportunity to explore novel pathways. In contrast, teams without star inventors often exhibit more balanced participation and a wider distribution of influence. This structure allows a broader range of ideas to surface and compete on merit, potentially enhancing the team’s overall cognitive flexibility and exploratory capacity.

In summary, the greater cognitive and social constraints faced by star inventors (compared to non-stars), amplified by their central team position, collectively hinder the team’s exploration of novel pathways, thereby reducing innovation novelty.

Therefore, we propose the following hypothesis:

*H1a*: Teams with star inventors are less likely to generate innovation of greater technological novelty.

At the subsequent phases following idea generation, the creative idea is further developed and implemented. According to the theory of innovation phases proposed by Perry-Smith and Mannucci (2017), support, influence, and shared vision, which are precisely the strengths of star inventors, become crucial determinants of innovation quality. First, the process of developing and implementing creative ideas involves a gradual elimination of uncertainty, which resonates with the motivation of stars having a conservative inclination. In this situation, star inventors are more willing to contribute their personal resources and influence to the team. Second, the extensive experience of stars aids the team in ensuring the feasibility and usability of creative ideas, tacit knowledge and even personal tricks of stars can help the team guarantee the execution of creative ideas (Singh and Fleming, 2010). Third, on the one hand, star inventors possess substantial social capital, which enables them to deploy resources to support team innovation and foster a conducive environment. On the other hand, the prominent visibility of star inventors within innovation networks signifies higher reputation and status, which in turn facilitates the acceptance of their opinions and improves their efficiency in deploying resources (Kehoe and Tzabbar, 2015). Moreover, their outcomes can gain wider exposure, thereby enhancing the dissemination and adoption of innovation output (Singh et al., 2016). Therefore, we propose the following hypothesis:

*H1b*: Teams with star inventors are more likely to generate innovation of greater technological impact.

#### 2.2.2 The moderating effect of technological turbulence

In reality, innovation teams are embedded within their surrounding systems. Changes in the external environment can trigger shifts in team members’ perceptions, leading to variations in individual behaviors (Burke et al., 2006; Yun et al., 2019). Technological turbulence refers to the uncertainty of the technological environment (Calantone et al., 2003). The iteration and update of knowledge occur more rapidly in a turbulent technological environment, and the applicability of existing expertise diminishes when addressing new situational demands (Song et al., 2005; Chen et al., 2018). Meanwhile, scholars in organizational management have suggested that new power-dependence orders within the team will emerge with the obsolescence and advancement of knowledge (Chen et al., 2015; Chen et al., 2018). A de-dependence process ensues, and team members will no longer rely heavily on the opinions and advice of star inventors as they did in a stable technological environment. In this situation, a new question arises about how the dual effect of star inventors on technological novelty and impact will vary in a turbulent technological environment.

As we have argued, the negative effect of star inventors on technological novelty is mainly due to their motivation to protect their status and the potential for an empiricism trap within their cognitive structure. These factors impede the development of cognitive flexibility. However, a more flexible cognitive structure is required to handle the high uncertainty inherent in a turbulent technological environment (Audia and Goncalo, 2007; Almandoz and Tilcsik, 2016; Chen et al., 2018). The mismatch between the capacities required for innovation and the actual actions of the actors is further exacerbated. This leads to a worsening of the impact of star inventors on technological novelty.

The potential silver lining is that the de-dependence resulting from technological turbulence may encourage non-star members to contribute more. This could, to some extent, foster innovative thinking and mitigate the negative impact that star inventors have on technological novelty. Nevertheless, compared to non-stars, star inventors have a more significant influence on a team. This suggests that when there is a conflict between the directions influenced by stars and non-stars, the overall outcome is likely to be dominated by the changes initiated by star inventors.

Based on the aforementioned reasoning, the negative impact of star inventors on the technological novelty of team innovation is likely to be intensified within a turbulent technological landscape. Consequently, we propose the following hypothesis:

*H2a*: Technological turbulence can enhance the negative effect of star inventors on technological novelty.

During the phases of developing and implementing creative ideas, star inventors leverage their resource advantage to ensure the success and expand the diffusion of innovation. Exactly, the extent to which the resource advantage of star inventors can promote the technological impact of innovation output depends on three factors: (1) the intrinsic value of the resources possessed by star inventors; (2) the willingness of star inventors to share these resources; and (3) the extent to which team members can absorb and utilize these resources (Tortoriello, 2015; Singh et al., 2016).

Regrettably, a turbulent technological environment is detrimental to all three aforementioned aspects. First, technological turbulence is characterized by the rapid obsolescence and advancement of knowledge (Chen et al., 2018). The existing expertise of star inventors may no longer be directly applicable to new situational demands (Calantone et al., 2003; Song et al., 2005). This leads to the fact that the intrinsic value of the resources held by star inventors diminishes in a turbulent technological environment. Second, the reduced value of stars’ expertise leads to the situation that non-star members’ expectation of stars’ performance will be damped down. Team members become less trusting and even become critical toward the decisions and advice provided by star inventors (Barton and Sutcliffe, 2009; Burkhardt and Brass, 1990). As a result, the team experiences increased cost and a decrease in efficiency when recombining star inventors’ expertise. Third, the decreased reliance and trust of team members in star inventors create a negative feedback loop that affects the willingness of star inventors to share their knowledge. This ultimately leads to a negative shift in the effect of star inventors on the technological impact of innovation output. Therefore, we propose the following hypothesis:

In summary, within the turbulent technological landscape, the value of star inventors’ expertise is likely to diminish, the cost of utilizing their resources is expected to increase, and their inclination to share may be weakened. Consequently, we propose the following hypothesis:

*H2b*: Technological turbulence can diminish the positive effect of star inventors on technological impact.

#### 2.2.3 The moderating effect of internal network cohesion

Team innovation is a process of complex interaction among team members (Li et al., 2018; Li et al., 2020). The pattern of interaction among team members is crucial for leveraging the influence of star inventors. We further argue that the internal network cohesion of the team serves as a valuable contextual factor that mitigates the drawbacks and amplifies the advantages of star inventors.

From a social networks standpoint, network cohesion refers to the general level of “inclusiveness” (Scott, 2000) and “connectivity” (Wasserman and Faust, 1994) of a network. A cohesive network, which is a typical closed social structure, occurs when team members have dense and overlapping ties with each other (Freeman, 1978; Ibarra, 1993; Martí et al., 2017). Through frequent past collaboration, stronger trust and common knowledge are more likely to be established among team members (Reagans and McEvily, 2003; Obstfeld, 2005; Rost, 2011). This cohesive structure can develop a strong deterrence mechanism to individual misconduct and thus enhance the reciprocal expectations among members (Coleman, 1988; Fleming et al., 2007). Individual motivation to contribute to team outcomes will be correspondingly strengthened. Meanwhile, strong trust relationships and rich common knowledge embedded in a cohesive network are beneficial for promoting the sharing of knowledge and resources, especially for tacit, complex, and proprietary assets (Sorenson et al., 2006).

Specifically, the negative impact of star inventors on technological novelty stems primarily from constrained cognitive flexibility at the idea generation stage. Teams with high internal network cohesion can foster a psychologically safe environment, empowering members to critically evaluate individual viewpoints. In a context of heightened mutual familiarity and trust, team members demonstrate greater willingness to vocalize their perspectives. The team demonstrates reduced reliance on the creative ideas of star inventors in this situation. Team members will provide constructive feedback and effective refinements once star inventors propose too conservative ideas. The risks caused by the cognitive rigidity of star inventors will be mitigated. The opportunity to generate more novel ideas will be promoted.

Moreover, the positive impact of star inventors on technological impact can be further strengthened. The extent to which the team can benefit from the precious expertise and resources of star inventors depends on two aspects: the star inventors’ willingness to share and the extent to which the team can assimilate. This is precisely the advantage of a highly cohesive network. First, competition and cooperation simultaneously exist among team members. Knowledge serves not merely as the raw material for innovation but also as personal competitive advantages (Yang and Wu, 2008). Individuals will exhibit a stronger willingness to share only within a team characterized by mutual trust. Second, star inventors’ expertise and technical know-how is highly tacit, common knowledge embedded in a cohesive network provides a superior environment for transmission. The efficiency of assimilating for the team will be enhanced in this situation. Therefore, we propose the following two hypotheses on the contextual effect of internal network cohesion:

*H3a*: Internal network cohesion can diminish the negative impact of star inventors on technological novelty.

*H3b*: Internal network cohesion can enhance the positive impact of star inventors on technological impact.

## 3 Materials and methods

### 3.1 Data

Patent data collected from the U. S. Patent and Trademark Office (USPTO) is used to test our hypothesis in this research. The intensification of market competition necessitates the protection of intellectual property rights for the majority of technological innovations, often achieved through patents. Patents entail a thorough and meticulous examination, thereby ensuring the reliability of the information provided. Patent documents encompass abundant valuable information, such as inventors, classification, dates, citations, and so on. These details greatly facilitate the tracing of innovative activities and outcomes of the research team in this study. Our data processing proceeded in two main stages:

First, we identified star inventors on a yearly basis. Using a rolling five-year window (from year *t*-5 to *t*-1), we aggregated patent data within each industry. From these patents, we generated a comprehensive list of all inventors and identified every patent associated with each inventor during the five-year window. For every inventor, we then calculated two key metrics: the total number of patents they contributed to (quantity) and the aggregate citation count these patents received (quality). Inventors were then ranked within their respective industries based on these dual criteria. Those performing in the top 5% of both quantity and quality within their industry were formally designated as star inventors for year *t*.

Second, by using the common approach in patent-based research, all inventors listed on a patent are treated as a collaborative team (Wuchty et al., 2007; Singh and Fleming, 2010). For each team of one patent in year *t*, we identified all members and determined whether any had been labeled as star inventors based on their historical performance up to year *t*-1. Team-level variables were then constructed using the historical patent portfolios of all members.

Given the substantial computational demands, especially in matching citation data, we conducted data preprocessing and variable construction using MATLAB. The processing was performed on a high-performance workstation equipped with 96 GB of RAM to ensure efficient handling of the large-scale data operations.

To ensure the generalizability of this study, patent data from the USPTO spanning the years 1985 to 2020 were collected. However, considering the need for an adequate period for observing characteristics of inventors and a sufficient lag for observing backward citations, our final sample consisted of 2,562,340 patent teams spanning 35 industries from 1990 to 2015. It is worth noting that, to mitigate potential biases arising from the differences between domestic and foreign patents, this study’s sample only includes patents developed by individuals or organizations in the United States.

Moreover, to trace inventors’ inventive experiences precisely, on the basis of the official disambiguation provided by USPTO, we apply the matching algorithm employed by Li et al. (2014) to identify unique inventors. On the other hand, the USPTO moved from using the United States Patent Classification (USPC) system to the Cooperative Patent Classification (CPC) from 2013. Patents applied after 2013 are part of our sample. Therefore, CPC rather than USPC is utilized as the foundational element for measuring knowledge characteristics to ensure data consistency.

### 3.2 Method

#### 3.2.1 Measurements of variables

##### 3.2.1.1 Dependent variables

We use two variables, namely, *Tech_Novelty* and *Tech_Impact*, to indicate the value of technological novelty and technological impact of innovations, respectively. Following the method frequently used in previous research, *Tech_Novelty* is measured by the number of new knowledge combinations (Fleming et al., 2007; Lee et al., 2015). The pairwise CPC four-digit code is used to represent knowledge combinations. A combination is considered new if it has not been observed in previous innovations by any of the team members. The greater the number of new combinations, the higher the level of technological novelty. Furthermore, the *Tech_Impact* variable in this study is measured using the number of forward citations, a widely accepted indicator of innovation value and ultimate success (Lee et al., 2015; Li et al., 2018).

##### 3.2.1.2 Independent variable

To investigate the effect of star inventors on team innovation, we define a dummy variable named *Star_Effect* that serves as an indicator of whether the focal team comprises star inventors. This variable takes on a value of 1 when there is at least one star involved in the innovation. Following the well-established way, star inventors are identified as those who simultaneously demonstrate high levels of both quantity and quality in their innovation output (Ernst and Vitt, 2000; Liu et al., 2018).

Meanwhile, the selection of star inventors is tailored to each specific industry due to potential variations in output distribution across different industries. For each industry, we extract all patents associated with each inventor and quantify both the number of patents and the number of forward citations received. Each inventor is labeled as a star if he/she achieves a ranking within the top 5% in both the aforementioned metrics.

##### 3.2.1.3 Moderating variables

###### 3.2.1.3.1 Technological turbulence (*Tech_Turb*)

Following the methods widely adopted in previous studies, technological turbulence is measured by the rate of technological changes by technological field in each year (Yun et al., 2019; Dai et al., 2018; Goerzen, 2007; Lavie, 2006; Luque, 2002). For each team, their technological fields are uniquely identified as the first four digits of the CPC code. To obtain the technological turbulence of each technological field, we compute the number of all patent applications in the focal technological field between periods *t* and *t*−1. The percentage change in these two numbers is used to reflect the extent of technological turbulence. Algebraically, the variable *Tech_Turb* indicating technological turbulence is calculated by the following formula:


$$\text{Tech}\_\text{Turb}=\frac{N_{\mathrm{it}}-N_{i(t-1)}}{(N_{\mathrm{it}}+N_{i(t-1)})/2}$$

Where *N_it_* is the number of patent applications in technological field *i* during the *t* period.

###### 3.2.1.3.2 Internal network cohesion (*Inter_Cohesion*)

The literature on social networks suggests that dense network structures and strong network ties are both beneficial for improving network cohesion (Coleman, 1988; Martí et al., 2017). Therefore, we utilize internal network density, weighted by the strength of ties, as a metric to gauge the level of internal network cohesion. Tie strength, in this context, represents the frequency of collaboration between two inventors over the past 5 years. The specific formula of internal network cohesion is as follows:


$$\text{Inter}\_\text{Cohesion}=(\sum_{i=1}^{n-1}\sum_{j=i+1}^{n}d_{\mathrm{ij}}s_{\mathrm{ij}})/(\frac{n(n-1)}{2})$$

where *n* is the number of team members, *d_ij_* presents whether inventor *i* and inventor *j* have collaborated (*d_ij_ = 1*) or not (*d_ij_ = 0*) in the past 5 years. *s_ij_* is the strength of the tie between inventor *i* and inventor *j* correspondingly.

##### 3.2.1.4 Control variables

Eight control variables are used to control the potential impact of patent and team characteristics. At terms of team characteristics, three variables including *Team_Size, Nostar_Ex*, *Govern_inter* are controlled. *Team_Size* refers to the number of team members, *Nostar_Ex* is measured by the number of patents invented by Non-star members, *Govern_inter* represents the fact that whether the team is funded by government. At terms of patent characteristics, five variables including *Claims, Tech_Scope, Patent_Citation, Science_Ref, Foreign_Citation* are controlled. *Claims* means the number of claims in patent specification. *Tech_Scope* is measured by the number of four digital CPC codes involved by the focal patent. *Patent_Citation* is measured by the number of backward patent citations. *Science_Ref* indicates the number of scientific references cited by the focal patent. *Foreign_Citation* is the number of foreign patents cited by the focal patent.

Moreover, in order to control the difference in technological outputs resulting from different time period, assignee type, and industry, three dummy variables indicating granted year, assignee type^1^, and industry^2^ are also included in our model.

#### 3.2.2 Model specification

The dependent variables *Tech_Novelty* and *Tech_Impact* indicate the number of new knowledge combinations and forward citations, respectively. A Laplace transformation is used before regression analysis to adjust the corresponding distribution. Therefore, the value of the dependent variable in the following analysis is the log transformation based on one plus the original value. Meanwhile, robust rather than normal standard error is used in this research to control for the potential bias due to heteroscedasticity (Greene, 2003).

Table 1 provides the descriptive statistics of all variables used in this research. Table 2 describes the correlation between pairwise variables. Apart from a few exceptions, most correlation coefficients are less than 0.1. The variance inflation factor (VIF) of all independent variables are also calculated. The VIF of star_effect is 1.13, while the VIFs for most control variables were below 2, with a mean VIF of 1.28. The results show that VIF scores are significantly far from the generally accepted thresholds (10 for a single variable and 4 for the average), regardless of whether the VIF scores are single or average. We also conduct stepwise regression to test stability in coefficient estimates when variables are added or removed. The results show that the coefficients remain stable in both value and significance across various model specifications. Therefore, multicollinearity does not appear to be a considerable issue.

**Table 1:** Descriptive statistics.

No.	Variable	Observations	Mean	SD	Min	Max
1	*Tech_Novelty*	2,562,340	0.9565	3.0075	0	277
2	*Tech_Impact*	2,562,340	20.6974	61.5854	0	4,318
3	*Star_Effect*	2,562,340	0.2232	0.4164	0	1
4	*Tech_Turb*	2,562,340	0.0500	0.1629	−1.69	2
5	*Inter_Cohesion*	2,562,340	1.5283	8.2719	0	800
6	*Team_Size*	2,562,340	2.5588	1.8269	1	76
7	*Nostar_Ex*	2,562,340	5.4598	8.1582	0	288
8	*Claims*	2,562,340	18.4937	12.8866	1	887
9	*Patent_Citation*	2,562,340	23.3834	69.1166	0	5,841
10	*Science_Ref*	2,562,340	7.9246	35.0569	0	2,964
11	*Foreign_Citation*	2,562,340	4.4223	21.7944	0	2,390
12	*Tech_Scope*	2,562,340	2.0156	1.3099	1	25
13	*Govern_Inter*	2,562,340	0.0372	0.1893	0	1

**Table 2:** Correlation matrix.

No.	1	2	3	4	5	6	7	8	9	10	11	12
2	0.008*											
3	−0.077*	0.081*										
4	0.014*	0.057*	0.002*									
5	−0.032*	−0.004*	0.235*	−0.008*								
6	−0.008*	0.024*	0.234*	−0.002*	0.106*							
7	−0.055*	−0.068*	0.087*	−0.036*	0.071*	0.420*						
8	0.016*	0.102*	0.091*	0.007*	0.033*	0.089*	0.023*					
9	0.012*	0.124*	0.149*	−0.004*	0.068*	0.076*	−0.004*	0.078*				
10	0.004*	−0.013*	0.037*	−0.001	0.023*	0.066*	0.035*	0.021*	0.060*			
11	0.020*	0.117*	0.112*	−0.004*	0.053*	0.102*	0.032*	0.049*	0.730*	0.076*		
12	0.619*	0.012*	0.040*	0.007*	0.031*	0.086*	0.037*	0.028*	0.076*	0.047*	0.087*	
13	0.029*	−0.011*	−0.031*	−0.005*	−0.013*	0.040*	−0.010*	0.002*	−0.026*	0.047*	−0.007*	0.065*

The numbers correspond to the variable number in Table 1. * represents for *p* < 0.01.

## 4 Results

### 4.1 Results on the direct effects of star inventors

The direct effects of star inventors are investigated from two dimensions, including the effect of star inventors on technological novelty and that on technological impact. Table 3 shows the related results.

**Table 3:** Regression results on direct effect of star inventors.

Dependent variable	MODEL 1	MODEL 2	MODEL 3	MODEL 4
	*Tech_Novelty*		*Tech_Impact*	
*Team_Size*	−0.0117^***^	−0.000455^*^	0.0692^***^	0.0571^***^
	(−57.59)	(−2.24)	(151.00)	(124.68)
*Nostar_Ex*	−0.00860^***^	−0.00897^***^	−0.0134^***^	−0.0130^***^
	(−196.81)	(−201.39)	(−148.18)	(−144.62)
*Claims*	0.000116^***^	0.000431^***^	0.0133^***^	0.0130^***^
	(4.25)	(16.18)	(154.44)	(153.35)
*Patent_Citation*	−0.000628^***^	−0.000465^***^	0.00215^***^	0.00198^***^
	(−53.21)	(−44.58)	(60.82)	(58.84)
*Science_Ref*	−0.000151^***^	−0.000126^***^	0.000235^***^	0.000208^***^
	(−15.06)	(−12.98)	(10.55)	(9.47)
*Foreign_Citation*	0.000154^***^	0.000126^***^	0.000558^***^	0.000587^***^
	(3.94)	(3.70)	(5.98)	(6.65)
*Tech_Scope*	0.324^***^	0.327^***^	0.0830^***^	0.0793^***^
	(452.98)	(467.76)	(139.72)	(134.11)
*Govern_Inter*	0.0232^***^	0.00627^***^	−0.0410^***^	−0.0229^***^
	(12.09)	(3.33)	(−10.85)	(−6.08)
*Star_Effect*		−0.241^***^		0.257^***^
		(−309.96)		(135.16)
Constant	0.187^***^	0.123^***^	0.694^***^	0.763^***^
	(76.04)	(51.39)	(155.40)	(171.68)
Year Effect	Included	Included	Included	Included
Industry Effect	Included	Included	Included	Included
Assignee Type Effect	Included	Included	Included	Included
*N*	2,562,340	2,562,340	2,562,340	2,562,340

*t* statistics in parentheses.

*p < 0.05, **p < 0.01, ***p < 0.001.

In terms of the effect of star inventors on technological novelty, we begin with the baseline model (Model 1), which takes *Tech_Novelty* as dependent variable and includes all control variables. Thereafter, *Star_Effect* variable, indicating the fact that whether star inventors in team, is introduced in Model 2. The result shows that the effect of *Star_Effect* is negative and significant (*β* = −0.241, *p* < 0.001). It means that innovations generated by teams with star inventors is less novel in compared with those generated by teams without star inventors. Thus, Hypothesis 1a is supported.

An analogous process is utilized to test the effect star inventors on technological impact. The baseline model (Model 3), taking *Tech_Impact* as the dependent variable and including all control variables, is first provided. The independent variable *Star_Effect* is then introduced (Model 4). The result shows that the effect of *Star_Effect* is positive and significant (*β* = 0.257, *p* < 0.001). It implies that innovations generated by teams with rather than without star inventors will be more frequently used by others and thus have greater technological impact. Thus, Hypothesis 1b is supported.

Therefore, the effect of star inventors is not uniformly beneficial but rather presents a dichotomous outcome for the team, encapsulating both favorable and unfavorable consequences. While the joining of star inventors can enhance the technological novelty of innovation, the potential empiricism of star inventors may impair team creativity to some extent. Innovation includes multiple processes, such as the generation, retention, development, and implementation of creative ideas. Our results imply that the greatest strengths of star inventors are manifested in the later three stages. While during the stage of idea generation, it may be a more judicious strategy for teams with star inventors to attenuate the dominance of star inventors and enhance the involvement of other team members.

### 4.2 Results on the moderating effect of technological turbulence

Table 4 shows regression results on the moderating effect of technological turbulence. Similar to the analysis on the direct effect of star inventors, the moderating effect is also divided into two parts. One aspect involves examining how technological turbulence moderates the effect of star inventors on technological novelty, while another aspect focuses on exploring the contingency of star inventors’ effect on technological impact.

**Table 4:** Regression results on moderating effect of technological turbulence.

Dependent variable	MODEL 5	MODEL 6	MODEL 7	MODEL 8
	*Tech_Novelty*		*Tech_Impact*	
*Team_Size*	−0.000477^*^	−0.000479^*^	0.0569^***^	0.0569^***^
	(−2.35)	(−2.36)	(124.13)	(124.12)
*Nostar_Ex*	−0.00895^***^	−0.00896^***^	−0.0129^***^	−0.0129^***^
	(−201.06)	(−201.08)	(−143.17)	(−143.19)
*Claims*	0.000429^***^	0.000428^***^	0.0130^***^	0.0130^***^
	(16.10)	(16.10)	(153.17)	(153.17)
*Patent_Citation*	−0.000465^***^	−0.000465^***^	0.00198^***^	0.00198^***^
	(−44.59)	(−44.59)	(58.85)	(58.84)
*Science_Ref*	−0.000126^***^	−0.000126^***^	0.000211^***^	0.000211^***^
	(−12.95)	(−12.95)	(9.60)	(9.60)
*Foreign_Citation*	0.000126^***^	0.000126^***^	0.000588^***^	0.000589^***^
	(3.70)	(3.70)	(6.68)	(6.68)
*Tech_Scope*	0.327^***^	0.327^***^	0.0786^***^	0.0786^***^
	(467.53)	(467.52)	(132.97)	(132.98)
*Govern_Inter*	0.00625^***^	0.00626^***^	−0.0231^***^	−0.0230^***^
	(3.32)	(3.32)	(−6.14)	(−6.13)
*Star_Effect (IV)*	−0.241^***^	−0.240^***^	0.258^***^	0.260^***^
	(−309.93)	(−287.86)	(135.43)	(130.18)
*Tech_Turb (M1)*	0.0196^***^	0.0217^***^	0.231^***^	0.239^***^
	(9.62)	(10.21)	(53.17)	(51.22)
*IV × M1*		−0.0129^*^		−0.0447^***^
		(−2.17)		(−3.69)
Constant	0.120^***^	0.120^***^	0.729^***^	0.728^***^
	(50.09)	(49.83)	(162.42)	(161.89)
Year effect	Included	Included	Included	Included
Industry effect	Included	Included	Included	Included
Assignee type effect	Included	Included	Included	Included
*N*	2,562,340	2,562,340	2,562,340	2,562,340

*t* statistics in parentheses.

*p < 0.05, **p < 0.01, ***p < 0.001.

Based on former models with *Tech_Novelty* as the dependent variable, the moderating variable *Tech_Turb* and its interaction with *Star_Effect* variable are successively introduced (Model 5 and Model 6). The results show that the coefficient of interaction terms is negative and significant (*β* = − 0.0129, *p* < 0.05). Given that the coefficient for *Star_Effect* is negative, the negative interaction term indicates that as the value of *Tech_Turb* increases, the negative effect of *Star_Effect* becomes more pronounced. In other words, under conditions of high technological turbulence, the inhibitory effect of star inventors on novelty is stronger compared to that under conditions of low technological turbulence. Thus, Hypothesis 2a is supported.

Similarly, based on former models with *Tech_Impact* as the dependent variable, Model 7 and Model 8 sequentially introduce *Tech_Turb* variable and its interaction with *Star_Effect* variable to investigate how technological turbulence moderates the effect of star inventors on technological impact. The results show that the coefficient of interaction terms is negative and significant (*β* = − 0.0447, *p* < 0.001). Considering the positive coefficient of *Star_Effect*, the negative interaction term suggests that as the *Tech_Turb* variable increases, the positive effect of *Star_Effect* gradually diminishes. In other words, under conditions of high technological turbulence, the beneficial effect of star inventors on technological impact becomes smaller compared to that under conditions of low technological turbulence. Thus, Hypothesis 2b is supported.

Overall, during periods of heightened technological turbulence, both in terms of technological novelty and impact, the impact exerted by star inventors tends to manifest in a progressively adverse trajectory, which calls for vigilance within the team.

### 4.3 Results on the moderating effect of internal network cohesion

As analyzed previously, the effect of star inventors varies with the internal network cohesion of the team they are affiliated with. Table 5 shows the regression results on the moderating effect of internal network cohesion.

**Table 5:** Regression results on moderating effect of internal network cohesion.

Dependent variable	MODEL 9	MODEL 10	MODEL 11	MODEL 12
	*Tech_Novelty*		*Tech_Impact*	
*Team_Size*	−0.0000676	−0.000931^***^	0.0579^***^	0.0577^***^
	(−0.33)	(−4.57)	(126.37)	(125.92)
*Nostar_Ex*	−0.00889^***^	−0.00630^***^	−0.0129^***^	−0.0123^***^
	(−196.08)	(−138.23)	(−141.88)	(−128.25)
*Claims*	0.000443^***^	0.000409^***^	0.0130^***^	0.0130^***^
	(16.67)	(15.53)	(153.51)	(153.54)
*Patent_Citation*	−0.000453^***^	−0.000430^***^	0.00200^***^	0.00200^***^
	(−43.77)	(−42.32)	(59.10)	(59.14)
*Science_Ref*	−0.000124^***^	−0.000119^***^	0.000212^***^	0.000213^***^
	(−12.78)	(−12.50)	(9.66)	(9.70)
*Foreign_Citation*	0.000120^***^	0.000101^**^	0.000575^***^	0.000571^***^
	(3.51)	(3.02)	(6.51)	(6.46)
*Tech_Scope*	0.327^***^	0.329^***^	0.0797^***^	0.0800^***^
	(468.64)	(472.24)	(134.98)	(135.43)
*Govern_Inter*	0.00520^**^	0.00676^***^	−0.0249^***^	−0.0246^***^
	(2.76)	(3.64)	(−6.63)	(−6.54)
*Star_Effect (IV)*	−0.230^***^	−0.263^***^	0.278^***^	0.271^***^
	(−276.77)	(−307.03)	(141.38)	(133.82)
*Inter_Cohesion (M2)*	−0.00259^***^	−0.0506^***^	−0.00496^***^	−0.0151^***^
	(−31.91)	(−93.47)	(−38.23)	(−27.75)
*IV × M2*		0.0490^***^		0.0104^***^
		(90.11)		(18.56)
Constant	0.122^***^	0.106^***^	0.761^***^	0.758^***^
	(51.10)	(44.85)	(171.52)	(170.65)
Year effect	Included	Included	Included	Included
Industry effect	Included	Included	Included	Included
Assignee type effect	Included	Included	Included	Included
*N*	2,562,340	2,562,340	2,562,340	2,562,340

*t* statistics in parentheses.

*p < 0.05, **p < 0.01, ***p < 0.001.

Both Model 9 and 10 adopt *Tech_Novelty* as the dependent variable. The moderating variable *Inter_Cohesion* is introduced in Model 9 first. Model 10 added both the moderating term and its interaction term with the main variable simultaneously. The results show that the coefficient of the interaction term is positive and significant (*β* = 0.0490, *p* < 0.001), while the main effect of star inventors is negative (*β* = −0.263, *p* < 0.001). That is, for teams with a more cohesive network, the negative effect of star inventors will be weaker, and vice versa. Thus, Hypothesis 3a is supported.

Model 11 and Model 12 adopt *Tech_Impact* as the dependent variable. The coefficient of the main variable *Star_Effect* is positive and significant (*β* = 0.271, *p* < 0.001). The coefficient of the interaction term is also positive and significant (*β* = 0.0104, *p* < 0.001). It implies that for teams with a more cohesive internal network, involvement of star inventors will be more beneficial for improving the technological impact of innovation. Thus, Hypothesis 3b is supported.

Overall, contrary to the observed patterns on the varying effects of star inventors across different technological turbulence levels, teams characterized by a more cohesive internal network exhibit a capacity to mitigate the negative repercussions of star inventors while simultaneously enhancing the utilization of their inherent advantages.

### 4.4 Robustness check

#### 4.4.1 Identification of star inventors

Conceptually, star inventors are defined as inventors of remarkable innovation output of both high quantity and quality. We use the top 5% in the output ranking of both quantity and quality as the threshold to identify star inventors. In order to avoid potential bias in selecting star inventors, a larger (Top 10%) or a smaller (Top 1%) threshold is also used to repeat our analysis. The results show that there is no significant difference.

#### 4.4.2 Robustness of models

Dependent variables, including the number of new knowledge recombination and that of forward citations, are both count variable. A general OLS regression model with robust standard errors is used in our analysis to achieve better efficiency because of the sizable sample. A Laplace transformation is carried out before regressions. For such count data, we also use both the negative binomial regression (NBR) and zero-inflated NBR to observe potential bias due to the selection of the model. Results also show that there is no significant difference. Therefore, our results are robust on model specification.

#### 4.4.3 Measurement of variables

Technological impact is measured by the number of forward citations in the current analysis. We employed three alternative methods to measure technological impact to ensure the robustness of the analysis results. First, we measured the technological impact by considering only the forward citations received within 6 years after patent grant time. Second, we measured the technological impact by considering the number of forward citations after excluding self-citations. Third, we normalized the forward citation count for each patent by the average number of forward citations in the same technological field and in the same year. Models using three measurement yielded similar results, indicating no significant differences.

#### 4.4.4 Difference across industries

We have conducted additional subsample analyses by running separate regressions for each of the 35 industries. The results show that the core findings, concerning both the effects of star inventors on the technological impact and novelty of innovation, remain statistically significant across all industries.

## 5 Discussion and conclusion

### 5.1 Summary of findings

This research investigated the cross-level effect of star inventors on team innovation by evaluating innovation output from two perspectives: technological novelty and technological impact. The contingent effects of star inventors under different contexts of technological turbulence and internal network cohesion are also studied. The major findings are as follows:

First, the involvement of star inventors enhances the technological impact but diminishes the technological novelty of team innovation output. The greatest advantages of star inventors lie in their rich innovation experience, elevated reputation, and wide network exposure. However, these advantages also increase their risk of falling into the empiricism trap, leading to their tendency to maintain their own status and existing technological trajectories. These factors will come to play roles at various stages of innovation, thereby impacting innovation output across different dimensions, such as technological novelty and impact. The empirical results of this study validate this viewpoint.

Second, the impact of star inventors on team innovation worsens at higher levels of technological turbulence, regardless of their original positive or negative effect. In a turbulent technological environment, the pace of knowledge renewal and iteration accelerates, posing greater challenges to the existing R&D experience and knowledge systems of star inventors. On one hand, their experience becomes less applicable to new technological changes, limiting their positive role in the innovation process, while the destructive consequences of clinging to their established experience become more pronounced. On the other hand, non-star members experience heightened innovation motivation, leading to reduced trust in the experience of star inventors and limiting their ability to express opinions, resulting in potential loss of beneficial experiential knowledge transmission.

Third, a cohesive internal network is beneficial for the positive influence and suppression of the negative effects of star inventors. In a highly cohesive team, there is stronger trust among team members, and star inventors are more willing to share their personal experiences. Furthermore, there is more extensive expression of opinions within the team, allowing for thorough validation of complex technical details and minimizing information loss. The team’s ample expression of opinions also serves as a corrective mechanism for potential experiential biases that star inventors may possess.

### 5.2 Theoretical and practical contributions

This research has three main theoretical contributions. First, this research expands the literature on star inventors by moving beyond the prevalent emphasis on their positive impact on team innovation. Second, this research enriches the contextual factors, including the external environmental factors and internal team factors, exerting influences on roles played by star inventors. Third, this research endeavors to take one step forward in bridging three streams of research, namely, psychology, social networks, and knowledge management.

Our findings provide managers with a framework for leveraging star inventors by clarifying their impact on innovation (novelty vs. impact) and how these impacts are shaped by two key contingencies: technological turbulence and the internal cohesion. The central managerial challenge is not whether to have star inventors, but how to manage their involvement dynamically.

Our result that star inventors have a negative effect on novelty suggests that blind reliance on them during the idea-generation phase is counterproductive. To mitigate this, managers should deliberately create a psychological safety climate where non-star members feel empowered to contribute unconventional ideas without fear of immediate criticism from the star. More specifically, companies typically engage in two types of innovation: exploitation, which builds upon existing strengths, and exploration, which involves venturing into new territories. In the case of exploitative innovation, managers can assign leading roles to star inventors to maximize their experience and resource advantages. This will greatly benefit the strengthening of existing technological advantages. Conversely, during exploratory innovation, introducing anonymous brainstorming techniques to foster creativity among all inventors, while also tasking star inventors with the duty of identifying promising ideas and allocating resources to ensure the success of exploratory projects.

Our finding that technological turbulence strengthens the negative effect of star inventors on technological novelty implies that traditional reliance on star inventors’ past experience becomes riskier in fast-changing environments. Especially in the current era characterized by rapid advancements in AI technology, which pose significant challenges to established technological paradigms, enterprises must accelerate the renewal of their knowledge structures and team organizational models. Overreliance on traditional technical advantages and veteran experts may lead to insufficient sustainable innovative capacity. A more effective pathway to cope with accelerating knowledge iteration involves actively empowering early-career inventors while leveraging established star inventors to provide essential resources and social capital. Furthermore, our findings highlight that internal team cohesion facilitates positive spillovers among R&D members, indicating that a stable team serves as a resilient foundation against external volatility. This creates a clear decision-making rule for managers: under conditions of high technological turbulence, managerial priority should be given to identifying and nurturing emerging talent and constructing stable, trust-based team structures around them.

### 5.3 Limitations and future research

This study has several limitations that also offer avenues for future research. First, we utilize patent data to trace collaboration relationships and innovation performance. While patent data encompasses innovation information across various industries, which enhances the generalizability of our research, not all innovations result in patents. Second, we determine star inventors based on the quality and quantity of their innovation output. Nevertheless, there may be different categories of star inventors, such as those distinguished by network structure or social reputation. Moreover, while large-scale data allows us to mitigate some unobserved heterogeneity to a degree, the absence of numerous demographic characteristics results in the neglect of some intriguing factors, such as team diversity (e.g., gender, ethnicity) and internal power dynamics (e.g., whether the star researcher exhibits cooperative or dominant behaviors). A more in-depth examination of the role of star inventors and the impact of team contextual factors could be achieved by integrating survey or organizational data with patent records.

### 5.4 Conclusion

In conclusion, this research transcends the simplistic debate of whether star inventors are “good” or “bad” for teams. Instead, our findings confirm that star inventors have a dual effect: their involvement enhances a team’s innovation impact but simultaneously restricts its novelty. This duality is intensified by external environmental factors, technological turbulence amplifies the negative aspects of star influence while weakening the positive ones. Conversely, a cohesive internal network serves as a critical buffer, mitigating the negative effects and promoting the positive contributions of star inventors. By integrating insights from cognitive psychology and social capital theory, we present a more comprehensive picture: star inventors are not merely individual talents but pivotal social actors whose potential must be carefully harnessed through team construction and environmental design.

## Data Availability

The original contributions presented in the study are included in the article/Supplementary material, further inquiries can be directed to the corresponding authors.

## Footnotes

1 Five assignee types are included in our sample, such as Unassigned, United States Individual, United States Company or Corporation, United States Federal Government, and Foreign Company or Corporation.

2 Dummy variables indicating 35 industrial field are used in this research. Information on industry and assignee type of each patent is provided by the official data from USPTO.
