# Supplementary material for: Two sides of the same coin: the dual effect of star inventors on team innovation
Source: Front Psychol. 2026 Jan 6;16:1669363. doi: 10.3389/fpsyg.2025.1669363 (PMC12815762; doi:10.3389/fpsyg.2025.1669363)

**Appendix**

Patent data, which encompass a wide array of information such as application date, inventors, classification codes, industry categories, citation details, and assignees, have been extensively used in innovation-related research. The process of inventing patent represents a typical innovation activity. In line with common practice in the field, this study treats all inventors listed on a single patent as a collaborative team.

The core dependent variables in this study are the patent’s novelty and impact, while the presence of at least one star inventor in the team serves as the key independent variable. In addition, technological turbulence and internal network cohesion are included as moderating variables. The specific measurement methods for these variables are described below. (The measurement approach for technological turbulence is well-defined and has been clearly outlined in the main body of the paper; therefore, it will not be reiterated here.)

**Technological Impact (*****Tech_Impact*)**

Technological impact refers to a patent's influence on future technological development, which is commonly measured by its number of forward citations (cited by later patents). The underlying assumption is that a patent with more subsequent citations has exerted greater technological influence. Our raw data contain complete citation information for each patent. We thus quantify a patent's technological impact by counting how many times it appears in the citation records of later patents. In addition to the total number of forward citations, we also calculated the number of citations received by each patent within specific time windows (6 years in this study). These refined measures are widely adopted in patent-based innovation studies.

**Technological Novelty (*Tech_Novelty*)**

*Tech_Novelty* is measured by the number of new knowledge combinations ((Fleming et al., 2007; Lee et al., 2015). Pairwise CPC four-digit code is used to represent knowledge combinations. A combination is considered as new if it has not been observed in previous innovations by any of the team members. The greater the number of new combinations, the higher the level of technological novelty.

Suppose our data is as follows:

| Focal patent p | Inventors | Classification codes |
| --- | --- | --- |
|  | i1,i2 | c3, c4, c6 |

The focal patent has two inventors, i1 and i2, and belongs to three classification codes: c3, c4, and c6. We found all patents invented by inventor i1 and i2. Inventor i1 had invented two patents, p1 and p2, while inventor i2 had invented two patents, p3 and p4. Among these, patent p1 belongs to two 4-digit classification codes c1 and c2; p2 belongs to c1 and c3; p3 belongs to c2 and c5; and p4 belongs to c3 and c4.

| Inventor i1 | Previous Patents | | Classification codes | |  |
| --- | --- | --- | --- | --- | --- |
|  | p1 | | c1, c2 | |  |
|  | P2 | | c1, c3,c4 | |  |
| Inventor i2 | | Previous Patents | | Classification codes | |
|  |  | p3 | | c2, c5 | |
|  |  | P4 | | c3,c4 | |

First, we aggregate all previous patents of the team members. If two classification codes appear in the same patent, they are recorded as one combination having been previously combined. This allows us to identify all previously combined knowledge pairs from the team members' historical patents, as illustrated in Figure 1.

After the development of the new patent p, the set of integrated knowledge combinations changes. As shown in Figure 2, the combinations c3–c6 and c4–c6 did not appear in previous knowledge network. These newly emerged combinations are considered novel knowledge combinations, and the number of such new combinations is used to measure technological novelty of the focal patent.

| Figure 1 Previous knowledge combinations | Figure 2 Current knowledge combinations |
| --- | --- |
|  |  |

**Internal Network Cohesion (*Inter_Cohesion*)**

The literature on social networks suggests that dense network structures and strong network ties are both beneficial for improving network cohesion ((Coleman, 1988; Marti et al., 2017). Therefore, we utilize internal network density, weighted by the strength of ties, as a metric to gauge the level of internal network cohesion. Tie strength, in this context, represents the frequency of collaboration between two inventors over the past five years. The specific formula of internal network cohesion is as follows:

$$\text{Inter}\text{\_}\text{Co}\text{h}\text{esion}\text{=}\left( \sum_{\text{i}\text{=1}}^{\text{n}\text{−1}} \sum_{\text{j}\text{=}\text{i}\text{+1}}^{\text{n}} \text{d}_{\text{ij}}\text{s}_{\text{ij}} \right)/\left( \frac{\text{n}\left( \text{n}\text{−1} \right)}{\text{2}} \right)$$

where *n* is the number of team members, *d_ij_* presents whether inventor *i* and inventor *j* have collaborated (*d_ij_=1*)or not (*d_ij_=0*) in the past five years. *s_ij_* is the strength of the tie between inventor *i* and inventor *j* correspondingly.

Figure 3 Data Example

The specific steps of building internal team network are illustrated in Figure 3, 4, and 5. First, as shown in Figure 3, a rolling five years window is created. Inventors involved in the focal patent are considered as team members. All patents invented by these members in the past five years (t-1 to t-5) are used to observe the collaborative relationships previously.

After that, the mapping relationships between patents and inventors are extracted from patents in a certain time window (as shown in Figure 4). The co-patent relationship implying two inventors involve in the same patent is identified as the formal collaborative relationship between these two inventors. In the final collaboration network, the nodes represent inventors and the edges represent the collaborative relationships among inventors. The frequency of the situations that two inventors involve in the same patent is identified as the strength of the edge. The mapping relationships in Figure 4 can be finally reflected in the collaboration network shown in Figure 5. The internal network cohesion is measured by above mentioned formula.

Figure 4 Patent-inventor matching

Figure 5 Internal team network


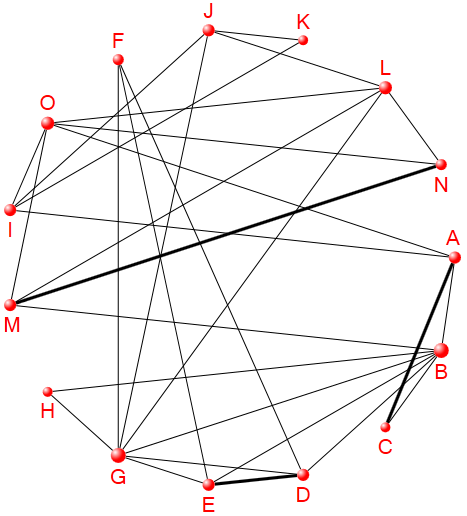

Supplement: Supplementary file 1 [file Table_1.DOCX]
